# The New Simulation of Quasiperiodic Wave, Periodic Wave, and Soliton Solutions of the KdV-mKdV Equation via a Deep Learning Method

**DOI:** 10.1155/2021/8548482

**Published:** 2021-11-26

**Authors:** Yong Zhang, Huanhe Dong, Jiuyun Sun, Zhen Wang, Yong Fang, Yuan Kong

**Affiliations:** College of Mathematics and Systems Science, Shandong University of Science and Technology, Qingdao 266590, China

## Abstract

How to solve the numerical solution of nonlinear partial differential equations efficiently and conveniently has always been a difficult and meaningful problem. In this paper, the data-driven quasiperiodic wave, periodic wave, and soliton solutions of the KdV-mKdV equation are simulated by the multilayer physics-informed neural networks (PINNs) and compared with the exact solution obtained by the generalized Jacobi elliptic function method. Firstly, the different types of solitary wave solutions are used as initial data to train the PINNs. At the same time, the different PINNs are applied to learn the same initial data by selecting the different numbers of initial points sampled, residual collocation points sampled, network layers, and neurons per hidden layer, respectively. The result shows that the PINNs well reconstruct the dynamical behaviors of the quasiperiodic wave, periodic wave, and soliton solutions for the KdV-mKdV equation, which gives a good way to simulate the solutions of nonlinear partial differential equations via one deep learning method.

## 1. Introduction

In recent decades, nonlinear partial differential equations (PDEs) are paid more and more attention in optical fiber communication, condensed matter physics, plasma physics, and fluid mechanics [1–3]. As one type of PDEs, the integrable equations attract more attention for their good properties; for example, the KdV-mKdV equation is widely used in nonlinear lattices, wave propagation of bound particles, acoustic waves, and thermal pulses [4–9]. In addition, the KdV-mKdV equation or the Gardner equation,

(1) $$\begin{array}{c} u_{t}+6uu_{x}+6u^{2}u_{x}+u_{xxx}=0, \end{array}$$

tends to the classical Korteweg–de Vries (KdV) equation or the mKdV equation in the limits of small or large waves, respectively. Via the complex Miura transformation,

(2) $$\begin{array}{c} v\left(x,t\right)=u\left(1+u\right)+i\frac{\partial u}{\partial x}, \end{array}$$

the KdV-mKdV equation will be transformed into the KdV equation:

(3) $$\begin{array}{c} \frac{\partial v}{\partial t}+6v\frac{\partial v}{\partial x}+\frac{\partial^{3}v}{\partial x^{3}}=0, \end{array}$$

which provides a model for describing surface and internal waves in oceans and the Rossby waves and can be also used to describe ion acoustic waves in plasmas and dust acoustic solitary structures in magnetized dusty plasmas [10–15]. It is usually used to describe the balance between nonlinear wave steepening and linear wave dispersion.

Via linear transformations of the function,

(4) $$\begin{array}{c} w\left(x,t\right)=\frac{1}{2}+u\left(x-\frac{3}{2}t,t\right), \end{array}$$

which is used to describe the acoustic waves in the plasma, the propagation of an elastic quasiplane wave in a lattice, electromagnetic waves in size-quantized films, and internal ocean waves under certain stratification [16–19]. Because PDEs contain more than two nonlinear terms or the lowest-order nonlinearity is cubic [4, 5, 20, 21] in many physics applications, the exact and numerical solutions of the KdV-mKdV equation can more visually depict the various phenomena of nature and help understand the physical mechanism of the phenomenon.

Recently, with the explosive growth of available data and computing resources, recent advances in machine learning and data analytics have yielded transformative results across diverse scientific disciplines, including image recognition, cognitive science, and genomics [22–25]. Machine learning techniques demonstrate impressive results for a range of highly complex tasks, especially where an accurate mathematical representation of the problem cannot be obtained [26, 27]. Therefore, the application of such techniques to solve PDEs will become a new subfield of machine learning. The physics-informed neural networks (PINNs) are introduced in [28, 29] and have become one of the most popular deep learning methods. PINNs employ a neural network as a solution surrogate and seek to find the best neural network guided by data and physical laws expressed as PDEs. A series of studies have shown the effectiveness of PINNs in fractional PDEs, stochastic differential equations, integrable systems, biomedical problems, and fluid mechanics [30–37]. Therefore, the deep learning method with underlying physical constraints is introduced to obtain the data-driven quasiperiodic wave, periodic wave, and soliton solutions of the KdV-mKdV equation in this paper.

The rest of this paper is organized as follows: Section 2 introduces the PINNs and briefly presents some problem setups.  Section 3 obtains data-driven quasiperiodic wave, periodic wave, and soliton solutions for the KdV-mKdV equation by the PINNs and discusses the effects of different numbers of initial points sampled, residual collocation points sampled, network layers, and neurons per hidden layer. Conclusion is given in the last section.

## 2. PINNs Method

In this section, the general architecture of PINNs is introduced and applied into the KdV-mKdV equation. Following notation similar to [33], the general form of the functions that the PINNs can approximate is

(5) $$\begin{array}{c} \frac{\partial u}{\partial t}=-N\left[u;\lambda \right],x\in \Omega ,t\in \left[0,T\right], \end{array}$$

where *u*(*t*, *x*) is the solution and *𝒩*[*u*; *λ*] is a nonlinear operator connecting the state variables *u* with the system parameters *λ*. The term *t* denotes time, and *x* denotes the system input. The domain Ω can be bounded based on prior knowledge of the dynamical system, and [0, *T*] is the time interval of system evolution. The model parameters *λ* can be constant or unknown. In case that *λ* is unknown, approximating function (6) becomes a problem of system identification, where we seek the parameters *λ* for which the expression in (6) is satisfied. In order to describe the physical laws of the dynamical system, the physics-informed neural network *f*(*t*, *x*) is defined as

(6) $$\begin{array}{c} f\left(t,x\right)=\frac{\partial u}{\partial t}+N\left[u;\lambda \right]. \end{array}$$

Note that if the system parameters *λ* are known, the nonlinear operator *𝒩*[*u*; *λ*] simplifies to *𝒩*[*u*]. A neural network is used to predict *u*(*t*, *x*) based on the inputs *t* and *x*. To determine *f*(*t*, *x*), automatic differentiation [38] of the components of the neural network *u*(*t*, *x*) is used. Based on this, the required derivatives of *u*(*t*, *x*) with respect to time *t* and system inputs *x* are computed. As a result, the neural network *f*(*t*, *x*) has the same parameters compared to the neural network *u*(*t*, *x*), but different activation functions. The shared parameters of these two neural networks are optimized by minimizing the loss function:

(7) $$\begin{array}{c} \text{MSE}={\text{MSE}}_{u}+{\text{MSE}}_{f}, \end{array}$$

where MSE_*u*_=(1/*N*_*u*_)∑_*i*_^*N*_*u*_^|*u*(*t*_*u*_^*i*^, *x*_*u*_^*i*^) − *u*^*i*^|^2^ denotes the mean squared error loss corresponding to the initial data, *N*_*u*_ is the total number of training data, MSE_*f*_=(1/*N*_*f*_)∑_*i*_^*N*_*f*_^|*f*(*t*_*f*_^*i*^, *x*_*f*_^*i*^)|^2^ denotes the mean squared error at a finite set of collocation points, and *N*_*f*_ is the total number of collocation points. The number of collocation points and training data influence the prediction accuracy and the computational time to optimize the loss function. The error MSE_*u*_ enforces the boundary conditions of the independent variables *x*, and MSE_*f*_ enforces the physics of the dynamical system imposed by condition (5), i.e., it penalizes deviations of the predicted physical law. Given a training data set and known system parameters *λ*, the parameters (weights and biases) of the neural networks are obtained to minimize (7). If the parameters *λ* are unknown, the same objective is trained but the system parameters are regarded as additional variables.

In this paper, the L-BFGS algorithm is used to optimize all loss functions. The algorithm is a full-batch gradient-based optimization algorithm based on a quasi-Newton method [39]. All codes in this article are based on Python 3.7 and TensorFlow 1.15, and all numerical examples reported here are run on a Lenovo Legion Y7000P 2020H computer with 2.60 GHz 6-core Intel(R) Core(TM) i7-10750H CPU and 16 GB memory. The hidden layers of the neural network use hyperbolic tangent activation functions. All codes can be mainly refered to https://github.com/maziarraissi/PINNs.

## 3. Data-Driven Solutions to the KdV-mKdV Equation

In the following, we consider the (1 + 1)-dimensional KdV-mKdV equation along with initial-boundary value conditions:

(8) $$\begin{array}{c} \left\{\begin{array}{c} u_{t}+6uu_{x}+6u^{2}u_{x}+u_{xxx}=0,x\in \left[0,0.5\right],\;t\in \left[-10,10\right],\\ u\left(t_{0},x\right)=a,\\ u\left(t,0\right)=u_{1}\left(t\right),u\left(t,0.5\right)=u_{2}\left(t\right), \end{array}\right. \end{array}$$

where *a* is the constant and *u*_1_(*t*) and *u*_2_(*t*) are certain boundary functions.

Now, *f*(*t*, *x*) is defined as

(9) $$\begin{array}{c} f≔u_{t}+6uu_{x}+6u^{2}u_{x}+u_{xxx}. \end{array}$$

The quasiperiodic wave, periodic wave, and soliton solutions of the KdV-mKdV equation have been obtained by many different methods, such as variational iteration method [40], tanh-function method [41], Jacobi elliptic function method [42–44], and so on. Here, the quasiperiodic wave, periodic wave, and soliton solutions are simulated by using the PINNs and compared with the known exact solutions, so as to prove the effectiveness of solving the numerical solutions *u*(*t*, *x*) by neural networks.

### 3.1. Data-Driven Quasiperiodic Wave Solution

Firstly, we introduce the quasiperiodic wave solution which have been derived the Jacobi elliptic function method in [44], and the quasiperiodic wave solution is formed as

(10) $$\begin{array}{c} u\left(t,x\right)=-\frac{1}{2}+k^{2}\text{cn}\left(kx+\left(\frac{3}{2}-2k^{4}+k^{2}\right)\left.kt\right],k\right), \end{array}$$

where cn( . ) is the elliptic cosine function and 0 ≤ *k* ≤ 1.

In order to numerically construct data-driven quasiperiodic wave solution of equation (8), quasiperiodic wave solution (10) is reduced to

(11) $$\begin{array}{c} u\left(t,x\right)=-\frac{1}{2}+\frac{81\text{cn}\left(\left(9/10\right)x+\left(44901/50000\right)t,\left(9/10\right)\right)}{100}, \end{array}$$

when *k* = 0.9. The corresponding initial condition is obtained by substituting a specific initial value into equation (11):

(12) $$\begin{array}{c} u\left(t,x\right)=-\frac{1}{2}+\frac{81\text{cn}\left(\left(9/10\right)x-\left(44901/5000\right),\left(9/10\right)\right)}{100}, \end{array}$$

with taking [*x*_0_, *x*_1_] and [*t*_0_, *t*_1_] as [0, 0.5] and [−10, 10], respectively.

The traditional finite difference model on even grids in MATLAB with initial data (12) is adopted to generate the training data. Specifically, by dividing space [0, 0.5] into 51 points and time [−10, 10] into 401 points, quasiperiodic wave solution *u*(*t*, *x*) is discretized into 401 snapshots accordingly. We subsample a small training dataset that contain initial-boundary subsets by randomly extracting *N*_*u*_=120 from original initial-boundary data and *N*_*f*_=10000 collocation points which are generated by LHS [45]. After giving a dataset of initial and boundary points, the latent quasiperiodic wave solution *u*(*t*, *x*) is successfully learned by using Python and TensorFlow [46] with 4 hidden layers and 60 neurons per hidden layer to tune all learnable parameters of the neural network and regulating the loss function (8). The model achieves a relative *𝕃*_2_ error of 7.880803e^−03^ in about 1441 seconds, and the number of iterations is 8080.

**Figure 1:**
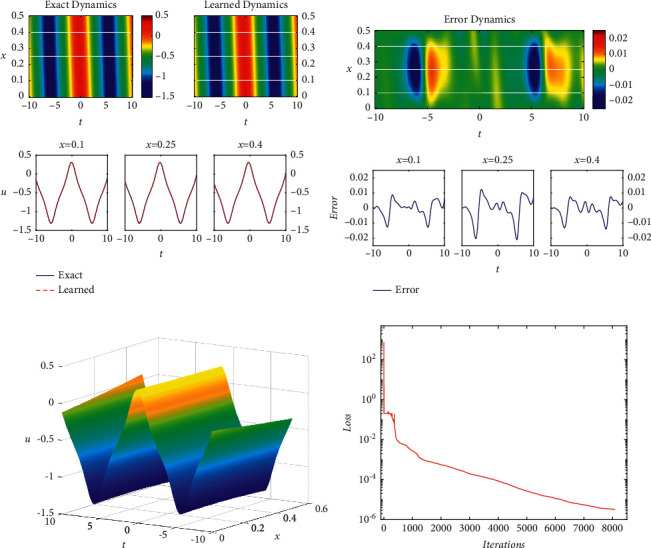
The quasiperiodic wave solution *u*(*x*, *t*): (a) the density diagram and profile diagrams of three different positions; (b) the error density diagram; (c) the three-dimensional diagram; (d) the loss curve.

Figure 1(a)shows the density diagrams of the quasiperiodic wave solution and clearly compares the exact solution with the learned spatiotemporal solution. The comparisons of different spatial locations *x* = 0.1, 0.25, and 0.4 are presented in the bottom panel of Figure 1(a).  Figure 1(b) presents the error diagram about the difference between the exact quasiperiodic wave solution. Combining with Figure 1(b), it is visible that the error between the numerical solution and the exact solution is very small. The error is mainly concentrated in the area where the solitary wave solution is generated; that is to say, the oscillation has a certain influence on the PINNs. The three-dimensional motion of the predicted solution and the loss curve at different iterations are given out in detail in Figures 1(c) and 1(d). From Figure 1(d), it is obvious that there are some obvious fluctuations in the training only at the start. The results show that the loss curve is very smooth when the number of iterations is more than 400, which proves the effectiveness and stability of the PINNs.

In addition, based on the same initial and boundary values of the quasiperiodic wave solution in the case of *N*_*u*_=120 and *N*_*f*_=10000, the control variable method is used to study the influence of different numbers of network layers and neurons per hidden layer on the quasiperiodic wave solution dynamics of the KdV-mKdV equation. The relative *𝕃*_2_ errors of different network layers and different neurons per hidden layer are given in Table 1. The relative *𝕃*_2_ errors with 4 network layers and 40 neurons per hidden layer when taking different numbers of subsampling points *N*_*u*_ in the initial-boundary data and collocation points *N*_*f*_ are shown in Table 2. From the data in Table 1, it can be seen that the number of network layers and single-layer neurons have no obvious regularity in the influence of the relative *𝕃*_2_ error and both have certain influence. However, the influence of the number of single-layer neurons is greater. To sum up, it is eyeable that the network layers and the single-layer neurons jointly determine the relative *𝕃*_2_ error to some extent. In the case of the same training data set, from Table 2, it is obvious that the influence of *N*_*u*_ on the relative *𝕃*_2_ error of the network is not obvious, which also indicates the network model with physical constraints can uncover accurate predicted solutions with smaller initial-boundary data and relatively many sampled collocation points.

### 3.2. Data-Driven Periodic Wave Solution

In this section, we study the periodic wave solution.

(13) $$\begin{array}{c} u\left(t,x\right)=-\frac{1}{2}+\frac{9}{25}\text{cn}\left(\frac{3}{5}x+\frac{6003}{6250}t,\frac{3}{5}\right), \end{array}$$

when *k* = 0.6 in equation (10). Then, [*x*_0_, *x*_1_] and [*t*_0_, *t*_1_] are taken as [0, 0.5] and [−10, 10], respectively. The corresponding initial condition is obtained by substituting a specific initial value into equation (14).

(14) $$\begin{array}{c} u\left(t,x\right)=-\frac{1}{2}+\frac{9}{25}\text{cn}\left(\frac{3}{5}x-\frac{6003}{625},\frac{3}{5}\right). \end{array}$$

The periodic wave solution is simulated numerically by using the same date generation and sampling method as the quasiperiodic wave solution. Then, *N*_*u*_=200 from original initial-boundary data and *N*_*f*_=1000 collocation points are sampled randomly. After training by Python and TensorFlow with 8 hidden layers and 20 neurons per hidden layer, the model achieves a relative *𝕃*_2_ error of 3.289852e^−03^ in about 873 seconds and the number of iterations is 7392.

**Figure 2:**
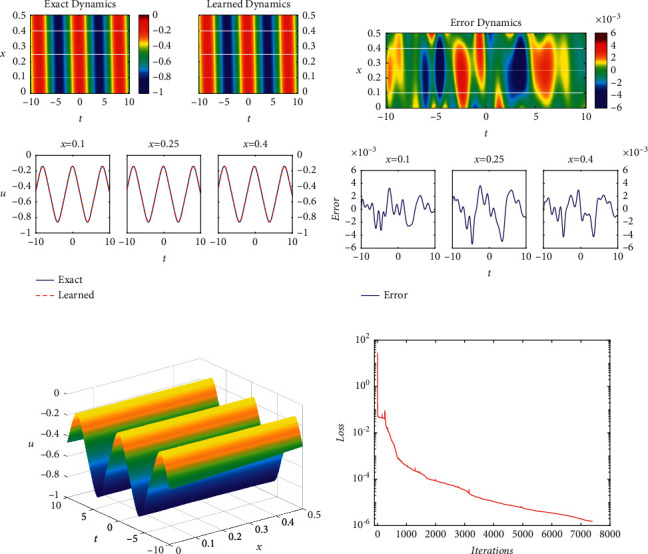
The periodic wave solution *u*(*x*, *t*): (a) the density diagram and profile diagrams of three different positions; (b) the error density diagram; (c) the three-dimensional diagram; (d) the loss curve.

Similar to Figure 1, Figure 2 shows the density diagrams, the error diagram about the difference between exact and learned periodic wave solutions, the three-dimensional figure, and the loss curve figure, respectively. In addition, the relative *𝕃*_2_ errors of different network layers and different neurons per hidden layer are given in Table 3. At the same time, the relative *𝕃*_2_ errors of different numbers of *N*_*u*_ and *N*_*f*_ in the case of 4 network layers and 40 neurons per hidden layer are shown in Table 4. By comparing with Table 1, it is found that the influence of the number of network layers and the single-layer neurons on the relative *𝕃*_2_ error is exactly the opposite. Obviously, combining Figure 1(b) and Tables 1 and2, it is eyeable that due to the simpler structure of the periodic solution, the relative *𝕃*_2_ error of the periodic solution is significantly smaller. At the same time, from Table 4, it is obvious that *N*_*u*_ and *N*_*f*_ have a certain influence on the relative *𝕃*_2_ error of the network, but the regularity is not obvious. From Figure 2(d), it is visible that there are some obvious fluctuations at the start and end of training, but they have little effect on the overall training, so the PINNs is still effective and stable.

### 3.3. Data-Driven Soliton Solution

Similar to Section 3.2, the soliton solution,

(15) $$\begin{array}{c} u\left(t,x\right)=-\frac{1}{2}+\text{sech}\left(x+\frac{1}{2}t\right), \end{array}$$

is obtained by equation (10) when *k* = 1. Then, the corresponding initial condition,

(16) $$\begin{array}{c} u\left(t,x\right)=-\frac{1}{2}+\text{sech}\left(x-5\right), \end{array}$$

is obtained with taking [*x*_0_, *x*_1_] and [*t*_0_, *t*_1_] as [0,0.5] and [-10,10], respectively.

The same date generation and sampling method as in Section 3.1 are used to simulate numerically the soliton solution. *N*_*u*_=120 from original initial-boundary data and *N*_*f*_=5000 collocation points are sampled randomly, which builds up a small training dataset. After giving a dataset of initial and boundary points, the latent quasiperiodic wave solution *u*(*t*, *x*) is successfully learned by Python and TensorFlow with 4 hidden layers and 40 neurons per hidden layer to tune all learnable parameters of the neural network and regulating loss function (7). The model achieves a relative *𝕃*_2_ error of 3.070442e^−03^ in about 82 seconds, and the number of iterations is 4119.

**Figure 3:**
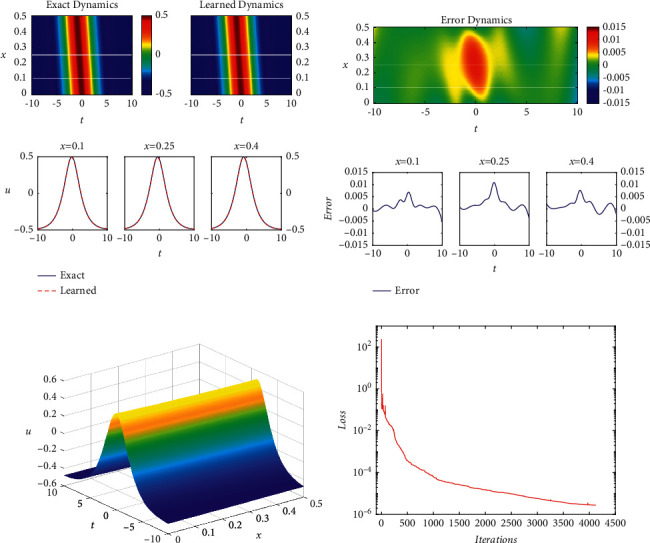
The soliton solution *u*(*x*, *t*): (a) the density diagram and profile diagrams of three different positions; (b) the error density diagram; (c) the three-dimensional diagram; (d) the loss curve.

Compared with the abovementioned figures and tables, it is obvious that the relative *𝕃*_2_ error of the soliton solution in Table 5 is larger than that of the quasiperiodic wave solution and smaller than that of the periodic wave solution. Similarly, the error is mainly concentrated in the place where the solitary solution is generated. At the same time, the influence of the number of network layers and the single-layer neurons on the relative *𝕃*_2_ error in Table 6 is similar to that of periodic wave solution. From Figure 3(d), it is visible that there are some obvious fluctuations only when the number of iterations is between 0 and 300 which proves the effectiveness and stability of the PINNs.

## 4. Conclusion

In this paper, the data-driven quasiperiodic wave, periodic wave, and soliton solutions of the KdV-mKdV equation are gained by the PINNs. Specifically, we discussed the influence of different numbers of network layers and neurons per hidden layer to solve the data-driven solutions of the KdV-mKdV equation. It is visible that the network layers and the single-layer neurons jointly determine the relative *𝕃*_2_ error to some extent and the error is mainly concentrated in the place where the solitary solution is generated. At the same time, according to the structures of the solutions, the applicability of the PINNs is also different. In other words, the more complex and regular the structure of the solutions, the better the effect of the PINNs. Remarkably, these results show that the PINNs can exactly recover different dynamical behaviors and obtain the data-driven solutions more quickly and efficiently for some nonlinear partial differential equations which cannot be solved by the traditional methods. Moreover, due to the physical constraints, the network is trained with just few data and has a better physical interpretability.

The PINNs obtain a series of results about various problems in the interdisciplinary fields of applied mathematics and computational science, which opens a new path for using deep learning to simulate unknown solutions and correspondingly discover the parametric equations. In this paper, we do not discuss the influence of noise on the neural network model, more complex boundary conditions, and more sampling methods. In the future work, we will focus on studying these problems to make the PINNs more universal and general.

## Figures and Tables

**Table 1:** The quasiperiodic wave solution of the KdV-mKdV equation: relative final learned error estimations in the *𝕃*_2_ norm for different numbers of network layers and neurons per hidden layer.

Layers	Neurons			
	20	40	60	80
2	3.602283*e* − 2	1.466837*e* − 2	1.780898*e* − 2	1.560210*e* − 2
4	9.527266*e* − 3	8.251322*e* − 3	7.880803*e* − 3	8.563890*e* − 3
6	8.885163*e* − 3	8.344743*e* − 3	8.318624*e* − 3	8.183045*e* − 3
8	9.012644*e* − 3	8.507917*e* − 3	8.578885*e* − 3	8.395397*e* − 3

**Table 2:** The quasiperiodic wave solution of the KdV-mKdV equation: relative final learned error measurements in the *𝕃*_2_ norm for different numbers of *N*_*u*_ and *N*_*f*_.

*N*_*u*_				
*N*_*f*_	80	120	160	200
5000	1.520107*e* − 2	3.494261*e* − 2	8.795864*e* − 3	9.298087*e* − 3
10000	8.991697*e* − 3	8.251322*e* − 3	9.737098*e* − 3	8.227559*e* − 3
15000	1.910283*e* − 2	1.097170*e* − 2	2.638983*e* − 2	8.782078*e* − 3
20000	1.358711*e* − 2	1.017950*e* − 2	9.225287*e* − 3	8.386114*e* − 3

**Table 3:** The periodic wave solution of the KdV-mKdV equation: relative final learned error estimations in the *𝕃*_2_ norm for different numbers of network layers and neurons per hidden layer.

Layers	Neurons			
	20	40	60	80
2	3.005433*e* − 2	2.388090*e* − 3	2.946261*e* − 2	2.826414*e* − 2
4	2.674150*e* − 2	3.407698*e* − 3	2.894121*e* − 2	3.767917*e* − 3
6	3.397864*e* − 3	3.812189*e* − 3	3.316271*e* − 3	3.612304*e* − 3
8	3.289852*e* − 3	1.668124*e* − 3	3.814982*e* − 3	4.327971*e* − 2

**Table 4:** The periodic wave solution of the KdV-mKdV equation: relative final learned error measurements in the *𝕃*_2_ norm for different numbers of *N*_*u*_ and *N*_*f*_.

*N*_*f*_	*N*_*u*_			
	80	120	160	200
5000	8.001687*e* − 3	7.878526*e* − 3	5.472321*e* − 3	4.188364*e* − 3
10000	6.470068*e* − 3	4.159932*e* − 3	4.096255*e* − 3	3.407698*e* − 3
15000	7.194250*e* − 3	4.526040*e* − 3	4.095404*e* − 3	3.566130*e* − 3
20000	1.824714*e* − 2	4.229454*e* − 3	6.915370*e* − 3	5.180385*e* − 3

**Table 5:** The soliton solution of the KdV-mKdV equation: relative final learned error estimations in the *𝕃*_2_ norm for different numbers of network layers and neurons per hidden layer.

Layers	Neurons			
	20	40	60	80
2	9.314110*e* − 3	7.436345*e* − 3	2.595096*e* − 2	1.772005*e* − 2
4	9.154403*e* − 3	3.070442*e* − 3	9.465375*e* − 3	9.040695*e* − 3
6	1.120311*e* − 2	7.512318*e* − 3	6.778210*e* − 3	5.110427*e* − 3
8	8.747611*e* − 3	5.329763*e* − 3	8.411414*e* − 3	8.499836*e* − 3

**Table 6:** The soliton solution of the KdV-mKdV equation: relative final learned error measurements in the *𝕃*_2_ norm for different numbers of *N*_*u*_ and *N*_*f*_.

*N*_*f*_	*N*_*u*_			
	80	120	160	200
5000	7.829712*e* − 3	3.070442*e* − 3	6.715074*e* − 3	4.582525*e* − 3
10000	9.306359*e* − 3	7.676940*e* − 3	6.886371*e* − 3	6.832231*e* − 3
15000	8.039516*e* − 3	7.248955*e* − 3	9.451375*e* − 3	5.664678*e* − 3
20000	6.343377*e* − 3	8.873952*e* − 3	5.490009*e* − 3	9.278955*e* − 3

## Data Availability

The data used to support the findings of this study are included within the article.
